# Heightened Delta Power during Slow-Wave-Sleep in Patients with Rett Syndrome Associated with Poor Sleep Efficiency

**DOI:** 10.1371/journal.pone.0138113

**Published:** 2015-10-07

**Authors:** Simon Ammanuel, Wesley C. Chan, Daniel A. Adler, Balaji M. Lakshamanan, Siddharth S. Gupta, Joshua B. Ewen, Michael V. Johnston, Carole L. Marcus, Sakkubai Naidu, Shilpa D. Kadam

**Affiliations:** 1 Neuroscience Laboratory, Hugo Moser Research Institute at Kennedy Krieger, Baltimore, Maryland, United States of America; 2 Department of Neurology and Developmental Medicine, Hugo Moser Research Institute at Kennedy Krieger, Baltimore, Maryland, United States of America; 3 Department of Neurology, Johns Hopkins University School of Medicine, Baltimore, Maryland, United States of America; 4 Department of Pediatrics, Johns Hopkins University School of Medicine, Baltimore, Maryland, United States of America; 5 Department of Biomedical Engineering, Whiting School of Engineering,Johns Hopkins University, Baltimore, Maryland, United States of America; 6 Sleep Center, The Children’s Hospital of Philadelphia, University of Pennsylvania School of Medicine, Philadelphia, Pennsylvania, United States of America; Oasi Institute for Research and Prevention of Mental Retardation, ITALY

## Abstract

Sleep problems are commonly reported in Rett syndrome (RTT); however the electroencephalographic (EEG) biomarkers underlying sleep dysfunction are poorly understood. The aim of this study was to analyze the temporal evolution of quantitative EEG (qEEG) biomarkers in overnight EEGs recorded from girls (2–9 yrs. old) diagnosed with RTT using a non-traditional automated protocol. In this study, EEG spectral analysis identified high delta power cycles representing slow wave sleep (SWS) in 8–9h overnight sleep EEGs from the frontal, central and occipital leads (AP axis), comparing age-matched girls with and without RTT. Automated algorithms quantitated the area under the curve (AUC) within identified SWS cycles for each spectral frequency wave form. Both age-matched RTT and control EEGs showed similar increasing trends for recorded delta wave power in the EEG leads along the antero-posterior (AP). RTT EEGs had significantly fewer numbers of SWS sleep cycles; therefore, the overall time spent in SWS was also significantly lower in RTT. In contrast, the AUC for delta power within each SWS cycle was significantly heightened in RTT and remained heightened over consecutive cycles unlike control EEGs that showed an overnight decrement of delta power in consecutive cycles. Gamma wave power associated with these SWS cycles was similar to controls. However, the negative correlation of gamma power with age (r = -.59; p<0.01) detected in controls (2–5 yrs. vs. 6–9 yrs.) was lost in RTT. Poor % SWS (i.e., time spent in SWS overnight) in RTT was also driven by the younger age-group. Incidence of seizures in RTT was associated with significantly lower number of SWS cycles. Therefore, qEEG biomarkers of SWS in RTT evolved temporally and correlated significantly with clinical severity.

## Introduction

RTT is a severe neurological disorder associated with mutations in the methyl-CpG-binding protein 2 gene (*MECP2*) resulting in the clinical manifestations of postnatal microcephaly, seizures, intellectual disability and respiratory irregularities [1–3]. The disorder occurs in one out of 10–22,000 females [2,4,5]. Disorders of sleep are a prominent feature of RTT and reportedly show variations with age and mutation type [6,7].The sleep problems frequently reported are decreased sleep time, longer latency to deep sleep (SWS) and fragmented sleep [8,9]. Research on RTT EEG has been focused on the impairments in sleep macrostructure and the associated respiratory parameters [10–12]. Studies examining the evolution of sleep in children using qEEG are scant [13] in general and nighttime polysomnography studies in patients with RTT have conflicting reports [11,12,14]. Qualitatively, patients with RTT have been shown to have decreased total sleep time, longer latency to sleep, and fragmented sleep, compared to controls [9] but severity differs with type of mutation and age [7].

Developmental changes in sleep architecture are well documented [15–17]. Meta-analysis [13] of quantitative sleep parameters have shown that the total sleep time, sleep efficiency, time spent in slow-wave-sleep (SWS), REM sleep and REM latency decreased with age [18]. The SWS sleep patterns in children have also showed temporal changes with advancing age [13,19,20] and these are known to be impaired in developmental disorders [21]. EEGs are currently being used in effectively identifying functional and cognitive biomarkers for many neurological disorders [22–24] and qEEG can yield objective biomarkers to help with patient management for better outcomes [25].

Little is known about the mechanisms by which *MECP2* modulates chronic sleep dysfunction and vice versa. In a recent study using a Mecp2-KO^Mecp2tm1.1Bird^ mouse model, we have reported the qEEG biomarkers of the associated severe sleep dysfunction in symptomatic Mecp2 null males [26]. The male KO mice showed significantly blunted delta power during SWS sleep cycles compared to their age-matched controls (i.e.; WT littermates). The translational value of studies in mouse models of RTT in general and newer conditional KO models in pre-clinical research is a subject of debate [27].Therefore, cross validation of findings between animal model studies and human studies is needed.

To investigate whether the qEEG related sleep dysfunction we reported for SWS sleep in the animal model of RTT [26] is also reflected in patients with RTT, we quantitated the SWS cycles in overnight EEGs from girls aged 2–9 yrs. with known *MECP2* mutations. We then correlated changes in SWS with age and clinical severity.

## Methods

The retrospective study consisted of 25 overnight EEGs acquired from girls aged 2–9 yr. old (RTT (n = 10) and non-RTT (n = 15). The EEGs acquired from girls with RTT (n = 10) had known mutations in *MECP2* and were recorded under guidelines (IRB # NA_00064949) approved by the Johns Hopkins Medicine IRB as baseline EEGs at the beginning of the clinical trial. Although the sample size of RTT patients is relatively small it is comparable to similar studies [28,29] in RTT due to the low incidence rates [i.e.;1in 10,000 to 20,000, [30]]. Informed written consent approved by the JHMIRB was obtained from a parent/guardian. De-identified EEG raw data were shared as per JHMIRB approved procedures. The EEGs from non-RTT age-matched girls were acquired from the Sleep Center at Children’s Hospital of Philadelphia and recorded during overnight polysomnography studies. The non-RTT girls (n = 15) were clinically referred to the Sleep Center for snoring but were otherwise healthy and reportedly also found to have normal polysomnography studies.

### EEG data acquisition

#### Clinical EEG raw data from Kennedy Krieger Institute

EEGs on girls with RTT were acquired as standard clinical overnight EEGs in the KKI Clinical Neurophysiology Laboratory, consistent with clinical EEG recording standards [31,32]. Recordings were performed using a standard 10–20 montage, on a Bio-logic machine (Natus Medical Incorporated, CA, USA), with recording at 256 Hz, with a bandwidth of 1–70 Hz using a forehead recording reference. Offline, EEG data were converted to European Data Format (EDF) and down-sampled to 128 Hz. Left F3, C3, and O1 channels with forehead recording reference underwent qEEG analysis. The RTT EEGs were recorded as baseline overnight EEGs as part of a pre-treatment workup for girls recruited into a drug trial study (NCT01520363). PSGs were not part of the study requirements. De-identified EEG raw data without age-related information exported as EDF files were handed over for further analysis with numerical IDs only.

#### Polysomnography derived EEGs raw data from Children’s Hospital of Philadelphia

EEGs were acquired overnight using the Rembrandt polysomnography system (Embla, Broomfield, CO) at 120 Hz using a forehead recording reference. Offline, EEG data were converted to European Data Format (EDF). The EEGs for control group came from PSG studies unlike the RTT group EEGs as described above. Controls were required retrospectively from the database. Control PSGs were performed overnight in the sleep laboratory. A Rembrandt polysomnography system (Embla, Broomfield, CO) recorded the following parameters: electroencephalographic leads (C3/A2, C4/A1, F3A2, F4A1, O1/A2, O2/A1), left and right electrooculograms, submental electromyogram (EMG) and tibial EMG, chest and abdominal wall motion using respiratory inductance plethysmography (Viasys Healthcare, Yorba Linda, CA), heart rate by electrocardiogram, arterial oxygen saturation (SpO2) by pulse oximetry (Masimo, Irvine, CA); end-tidal PCO2 (PETCO2), measured at the nose by infrared capnometry (Novametrix Medical System, Inc., Wallingford, CT), airflow using a 3-pronged thermistor (Pro-Tech Services, Inc., Mukilteo, WA) and nasal pressure by a pressure transducer (Pro-Tech Services, Inc., Walnut Cove, NC). Subjects were continuously observed by a polysomnography technician and were recorded on video with the use of an infrared video camera. Studies were scored (see S1 Table) using standard pediatric sleep scoring criteria [33]. Left F3, C3, and O1 channels with forehead recording reference underwent qEEG analysis the same as the RTT group. All EEGs in this study were assigned numerical identities and were analyzed blinded to age, group and mutation or clinical severity.

### Data analysis

EEGs were analyzed using non-conventional automated algorithms using R stats designed to quantitate high delta cycles similar to previously published pre-clinical studies in a mouse model of RTT [26]. Temporal evolution of qEEG data was done for both RTT and control groups as 2–5 yrs. olds and 6–9 yrs. olds. For this study, the 3 common channels (F3, C3, and O1) along AP axis with a forehead recording reference were analyzed for overnight EEGs from both de-identified group data sets.

Automated spectral analysis (Delta 0.5–4 Hz, Theta 5.5–8.5 Hz, Alpha 8–13 Hz, Beta 13–30 Hz and Gamma 35–45 Hz) using Sirenia sleep score module (Pinnacle Technologies Inc. KS, USA) calculated spectral power for every 10 sec epoch of the recorded overnight EEGs similar to previous animal model study [26]. The quantitated dataset were then exported into R-stats (http://www.r-project.org/). High delta cycles (i.e.; SWS) were identified as cycles with a delta power rate change of ≥10 mV/epoch over ≥100 epochs and represent NonREM sleep for these datasets. To quantitate the total power for each spectral category (i.e.; delta, gamma etc.) for the identified SWS cycles, area under curve (AUC) was calculated using trapezoidal summations in R-stats (http://www.r-project.org/). Theta/beta ratios (TBR) were calculated by dividing theta power at each epoch by its analogous beta power during SWS [34].

NonREM sleep cycles are usually further categorized as stages N1 to N3 in standard sleep scoring and are associated with increasing delta power. This well-characterized property of NonREM sleep was used to identify all SWS cycles using automated codes. The percent time of overnight recording spent in SWS was defined as SWS percent for this study. In addition, in order to calculate initial percentage rate of change of delta power for identified SWS cycles, delta power values per epoch were averaged for first one min. The same was done over a one min timeslot recorded 10 min later. A simple percentage change, which was the difference in the two calculated average numbers, divided by the first average was multiplied by 100 [% rate change Delta = (2nd value – 1st value)/1st value*100]. The percentage change in delta power over the entire duration of each SWS cycle was done similarly but the 2nd value was the calculated average of the last 1 min of the cycle over the first 1min. These quantitated values were also generated by an automated code written in R-stats software. REM sleep, described as “paradoxical wake” due to its qEEG signature similar to wake states, was not evaluated in this study.

#### Clinical severity scoring [see Tables 1, 2 and 3]

The baseline outcome measures for parameters quantitated in Table 1 are reported in Tables 2 and 3: a) Seizure frequency was measured by a seizure diary and interim evaluations by neurologists. All the medications that the girls with RTT were on during the time of their baseline EEG recording including anti-seizure medications are listed in Table 3. The RTT patients with anticonvulsant medications prescribed by their neurologists were allowed to continue on the same medications and doses throughout the study; b) Rett Syndrome Behavior Questionnaire (RSBQ); c) Pediatric Quality of Life Inventory (PedsQL version 4).

**Table 1:** Clinical severity score scale.

Severity Score (SS)	0	1	2	3
**Seizures**	Absent	Easily managed with meds	Managed with meds but occasional breakthrough	Recalcitrant seizures multiple meds
**Gait**	Normal	Mildly apraxic	Requires support for walking	Requires support to stand; wheelchair bound
**Scoliosis**	Absent	<20 degree	20–30 degrees	>30 degree, requires surgery
**Respiratory Irregularity**	Absent	Minimal BH	BH and HV > half the wake period	BH and HV > half wake period, ± cyanosis
**Hand Use**	Normal	Purposeful grasping	Tapping for needs	No hand use
**Speech**	Normal	Sentences/phrases	Single words	Non-verbal
**Sleep**	Normal	Awakens but falls back to sleep	Fragmented night sleep with daytime sleepiness	Unable to sleep through the night

BH = breath holding; HV = hyperventilation

**Table 2:** Clinical severity scores.

Subject ID	Age	Mutation	Language	Hand Use SS	Sleep SS	SZ	Respiratory Irregularity	Scoliosis	Ability to Walk	Total SS	SS
26	3y7mo	R294X	2	2	3	0	1	0	2	10	Moderate
27	3y10mo	R106C	2	2	0	0	0	0	1	5	Mild
28	6y1mo	D134C	3	2	1	0	0	0	0	6	Mild
29	8y8mo	R270X	3	3	0	0	0	2	3	11	Moderate
30	6y9mo	R133C	3	2	0	2	0	0	1	8	Moderate
31	5y9mo	R168X(P)	3	2	2	2	2	0	3	14	Moderate
32	7y3mo	1085 del_1197del	3	2	0	0	0	0	1	6	Mild
33	7y6mo	R133C	3	3	0	0	2	0	2	10	Moderate
34	9y11mo	R133C	1	2	0	1	0	1	1	6	Mild
35	3y10mo	T158M	3	2	1	0	2	0	1	9	Moderate

Severity Score = SS; SZ = seizures; Mild = 0–7; Moderate = 8–14; Severe = 15–21.

**Table 3:** Medications and EEG notes.

Subj ID	Age	Mutation	SZ	EEG notes	Medication
26	3y7mo	R294X	0	Sharp waves in central head regions on left, polyspikes bilateral and symmetrical	No medications
27	3y10mo	R106C	0	Rare sharp waves in central parietal regions on left	Depakote, Zantac,
28	6y1mo	D134C	0	No epileptiform discharges	MiraLax
29	8y8mo	R270X	0	Sharp waves in temporal-parietal regions bilaterally	Botox (paraspinal injections)
30	6y9mo	R133C	2	Sharp waves in central and central-temporal regions independently on left and right	Keppra, Levocarnitine
31	5y9mo	R168X(P)	2	Sharp waves in central-parietal regions bilaterally and independently on left and right	Depakote, Keppra, Baclofen,Diastat, Epipen, Prevacid, Glycolax
32	7y3mo	1085 del_1197del	0	Sharp waves in temporal-frontal regions bilaterally	Prevacid, Mitalax, Tums
33	7y6mo	R133C	0	Spikes waves present central parietal regions bilaterally and also independently on the left and right	Trazodone, Prevacid
34	9y11mo	R133C	1	Sharp waves present multifocally and slightly more prominent on right	Depakote, Miralax
35	3y10mo	T158M	0	Some sharp vertex waves	omega 3, Zantac, Miralax

SZ = seizures.

### Statistical analysis

All values are expressed as mean ± standard error (SE). Differences were calculated using independent sample t-tests between the RTT and control groups. Repeated measures within each group and between groups were evaluated using repeated measures AVOVAs and post-hoc Bonferroni’s for multiple pairwise comparisons. Significance was set at p<0.05.

## Results

### Age-matched RTT and control data sets

The study consisted of 10 overnight EEGs from girls with RTT aged 2–9 yr. old and 15 non-RTT control girls from the same age range. The average ages for RTT (n = 10) and non-RTT (n = 15) groups were 6.16±0.7 and 6.18±0.5 yrs. respectively and not significantly different from each other. The age-dependent temporal analysis done for the data sets for 2–5 vs. 6–9 yr. old were also not significantly different between the two groups. The average age was 4.1±0.35 vs. 3.9±0.66 yrs. for the younger 2–5 yr. old group and 7.9±0.24 vs. 7.6±0.46 yrs. for the older 6–9 yr. group between age-matched controls and RTT).

### SWS percent in overnight EEGs

**Fig 1:**
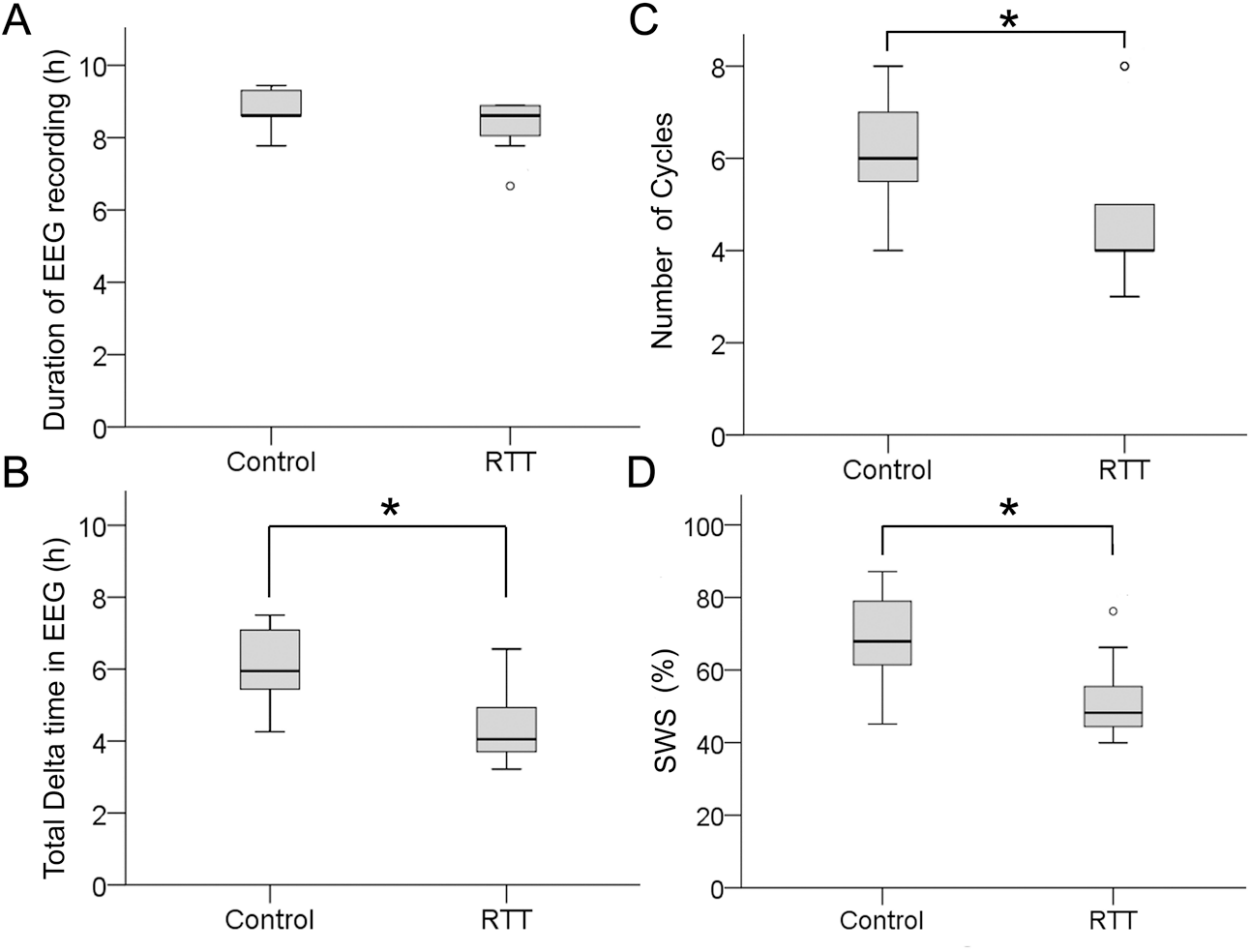
Boxplot of EEG spectral analysis and sleep structure analysis (A). Comparison of duration of overnight recordings in control EEGs with RTT EEGs revealed no significant differences. (B) Patients with RTT spent significantly less time during sleep in SWS (i.e.; high delta cycles) compared to the control group. (C) Patients with RTT had significantly fewer number of total SWS cycles compared to controls. (D) Therefore patients with RTT had significantly lower SWS percent.

Spectral analysis of overnight EEGs identified high delta SWS cycles (see methods). Duration of recorded overnight EEGs was not significantly different between the RTT and control group (control 8.83±0.14h vs. RTT 8.36±0.22h; Fig 1A). Total time spent in SWS over the entire duration of overnight EEGs was significantly lower (p = 0.0001; Fig 1B) in RTT (4.37±0.31h) compared to control EEGs (6.07±0.27h). The number of consecutive SWS cycles in the overnight EEGs was significantly lower (p = 0.015, Fig 1C) in RTT (4.70±0.58) compared to control EEGs (6.27±0.30), which was associated with significantly lower total time spent in SWS (Fig 1B). SWS percent, defined here as the percent time spent in SWS, was significantly (p = 0.002, Fig 1D) different between the two groups: control (69±3%) and RTT (52±3%). Therefore, overall there was a significant deficiency of time spent in SWS between patients with RTT and age-matched controls as a direct result of fewer SWS cycles in RTT overnight EEGs. Since overnight RTT EEGs in this study were not sleep-scored by conventional clinical standards (i.e.; certified sleep technician) for total sleep time and episodes of wakefulness, the custom automated analyses were restricted to the reliably identifiable SWS cycles.

### SWS cycle durations

**Fig 2:**
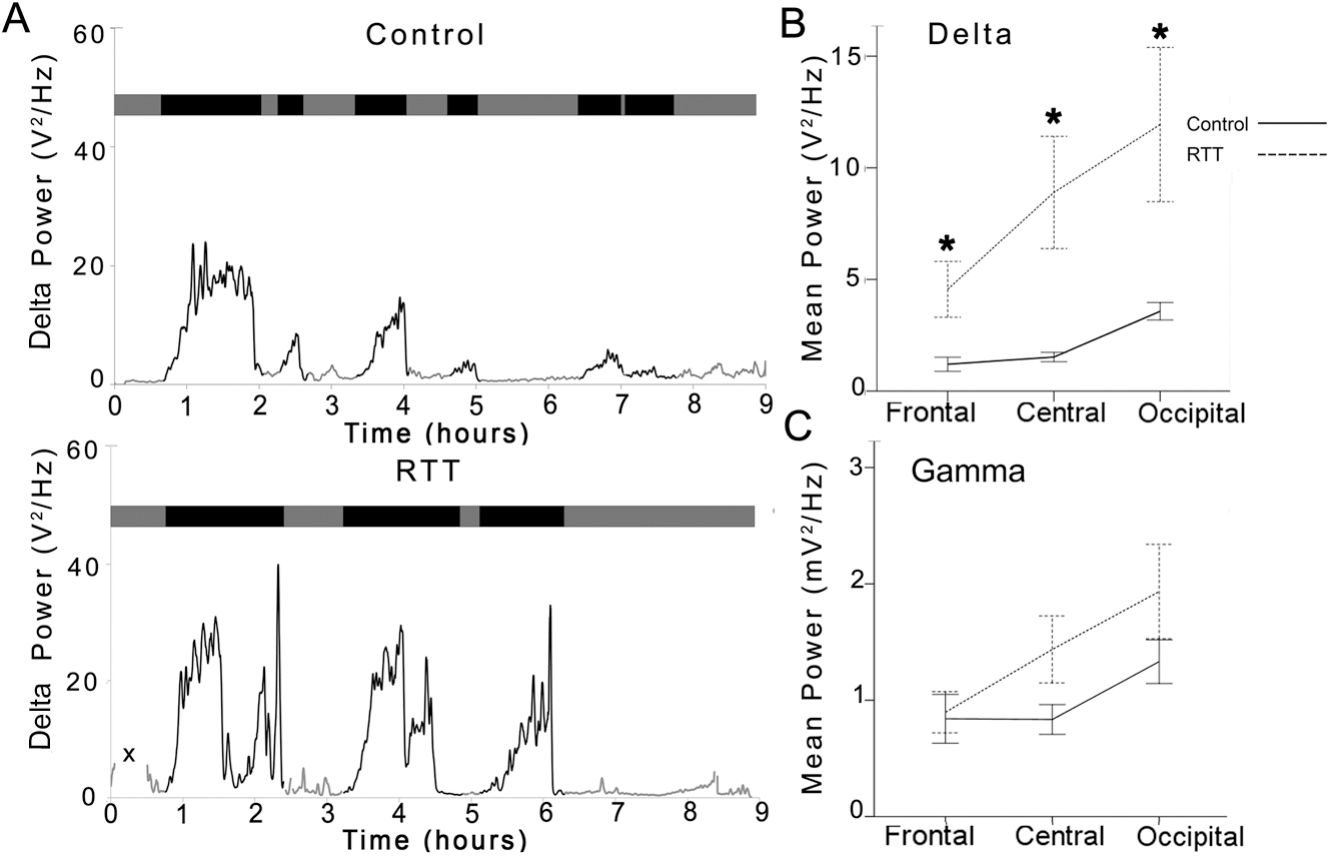
Comparison of control EEGs’ and RTT EEGs’ delta power. (A) Representative 8.5 hour EEG traces were scored as high delta power (black) and low delta power (grey). Comparison of RTT EEG with control EEGs revealed significantly higher delta power as well as fewer cycles. (B) RTT EEGs had significantly greater delta power in all three lead positions (frontal, central, occipital). (C) Patients with RTT had no significant difference in gamma power but revealed a trend of greater power reading in all three lead positions compared to control group.

SWS cycle durations were examined to evaluate temporal progression over the duration of the overnight recording. Average SWS cycle durations were not significantly different between RTT and control group (control .99±0.05h vs. RTT 1.12±0.09h). SWS cycles examined by age (2–5 vs. 6–9 yrs.) showed no significant differences between RTT and control group for average cycle durations either. The first two SWS cycles, at age group 2–5 years old did not show any significant difference between RTT and control group. However, in the 6–9 yr. olds, cycle 2 was significantly longer (p = 0.003) in patients with RTT (1.24±0.17h) compared to control patients (.61±0.08h). Repeated measures ANOVAs for analysis of consecutive SWS cycles for each group showed that the within subjects effects was significant for controls (F = 4.360, p = 0.01) that was absent in RTT (F = 0.705, p = 0.5). Analyzed by age group, this difference in within subjects effects for controls was driven by the 6–9 yrs. old group (F = 6.760, p = 0.004) and again absent in the RTT group. These data indicate that SWS cycle durations for consecutive SWS cycles in controls showed a significant trend in decline that was lost in RTT (Fig 2A). These SWS data match qEEG results reported for the KO Mecp2 mice^Mecp2tm1.1Bird^ both for fewer SWS cycles and light cycle (rodents are nocturnal) specific longer SWS cycle durations [26].

### Heightened delta power during SWS: AUC analysis

Automated codes written in R stats (http://www.r-project.org/) quantitated delta, gamma, beta, theta and alpha power AUCs within all the SWS cycles detected (Table 4). Control and RTT groups were found to have significant differences in the mean delta power for SWS cycles in all 3 channels quantitated (Fig 2) along the AP axis: F3 (p = 0.026), C3 (p = 0.017), and O1 (p = 0.039). The delta power in RTT EEG channels was significantly higher (Fig 2B; S1 Fig) than delta power in control EEG channels F3, C3, and O1 (Table 4 *). Along the AP axis, delta power showed significant increase in power in the occipital leads compared to the frontal (Fig 2B) in both the control [35] and RTT groups (repeated measures ANOVA, within-subjects effect, F = 38.02, p<0.0001 for controls and F = 10.62, p = 0.008 for RTT). Post-hoc pairwise comparisons showed that both C3 and O1 delta power was significantly higher than F3 in RTT (p = 0.02 for each pair) compared to controls where only O1 was significantly higher than F3 (p<0.0001). No significant differences were detected for AUCs for any other spectral power frequency analyzed between the two groups (see Table 4). Delta and gamma power oscillated reciprocally within each SWS cycle with delta power increasing and gamma power decreasing from the start to end of each SWS cycle in both groups similar to previously reported findings [36,37]. In addition, AUCs for gamma power also showed non-significant differences with similar trends along the AP axis in RTT group that were not significantly different from the control group during SWS sleep (Fig 2C, Table 4). Alpha power AUC was similar between RTT and control during SWS (Table 4) eliminating the possibility of alpha intrusion of delta cycles as an underlying cause of sleep disturbance [38]. Mean Theta and Beta power AUCs were not significantly different (Table 4) between groups during SWS nor were the Theta/beta ratios [34]. Thus, despite having significantly fewer SWS cycles during overnight EEG, RTT group of girls showed significantly heightened delta power during SWS compared their age-matched control group.

**Table 4:** Spectral power AUC during SWS sleep cycles.

**Whole Group**				
		**Power (mV^2/Hz)**		
		**F3 Delta**	**C3 Delta**	**O1 Delta**
**Genotype**	**Control**	1200.88±314.70 *	1523.95±207.77 *	3572.55±390.80 *
	**RTT**	4557.10±1250.39 *	8897.57±2513.32 *	11937.63±3457.57 *
		**F3 Alpha**	**C3 Alpha**	**O1 Alpha**
**Genotype**	**Control**	26.23±4.66	38.76±5.06	65.10±7.70
	**RTT**	43.52±16.18	73.71±25.26	99.18±37.83
		**F3 Gamma**	**C3 Gamma**	**O1 Gamma**
**Genotype**	**Control**	0.84±0.21	0.83±0.13	1.33±0.19
	**RTT**	0.90±0.18	1.44±0.29	1.93±0.41
		**F3 Beta**	**C3 Beta**	**O1 Beta**
**Genotype**	**Control**	10.42±2.34	11.68±1.44	19.01±1.56
	**RTT**	17.70±7.25	32.28±11.65	39.63±16.59
		**F3 Theta**	**C3 Theta**	**O1 Theta**
**Genotype**	**Control**	41.92±9.77	59.62±8.73	128.15±13.50
	**RTT**	72.65±23.19	118.45±38.16	168.08±49.71
**Age 2–5**				
		**Power (mV^2/Hz)**		
		**F3 Delta**	**C3 Delta**	**O1 Delta**
**Genotype**	**Control**	1785.84±568.70	1470.78±177.53	4128.71±553.93
	**RTT**	6322.10±2577.84	10751.07±5375.54	17529.32±6980.73
		**F3 Alpha**	**C3 Alpha**	**O1 Alpha**
**Genotype**	**Control**	34.97±8.41	38.20±8.74	63.84±13.68
	**RTT**	32.79±6.41	53.02±20.39	69.62±14.40
		**F3 Gamma**	**C3 Gamma**	**O1 Gamma**
**Genotype**	**Control**	2.77±1.09	1.74±0.34 #	2.45±0.28 #
	**RTT**	1.19±0.27	1.52±0.36	2.28±0.65
		**F3 Beta**	**C3 Beta**	**O1 Beta**
**Genotype**	**Control**	16.57±3.88	14.69±2.56	22.13±2.36
	**RTT**	9.68±3.87	20.42±9.53	21.63±6.27
		**F3 Theta**	**C3 Theta**	**O1 Theta**
**Genotype**	**Control**	64.83±17.00	71.09±16.62	155.16±23.52
	**RTT**	65.48±28.47	109.92±38.62	132.07±43.86
**Age 6–9**				
		**Power (mV^2/Hz)**		
		**F3 Delta**	**C3 Delta**	**O1 Delta**
**Genotype**	**Control**	689.04±220.51	1570.47±370.92	3085.91±519.71
	**RTT**	3380.43±1157.61	6486.93±2118.06	8209.84±3069.85
		**F3 Alpha**	**C3 Alpha**	**O1 Alpha**
**Genotype**	**Control**	18.59±3.22	39.25±6.20	66.21±9.02
	**RTT**	50.68±27.26	87.50±40.66	118.89±63.66
		**F3 Gamma**	**C3 Gamma**	**O1 Gamma**
**Genotype**	**Control**	0.35±0.04	0.55±0.06 #	0.86±0.06 #
	**RTT**	0.70±0.21	1.38±0.44	1.71±0.55
		**F3 Beta**	**C3 Beta**	**O1 Beta**
**Genotype**	**Control**	5.04±0.55	9.04±0.87	16.29±1.63
	**RTT**	23.05±11.71	40.18±18.42	51.63±27.17
		**F3 Theta**	**C3 Theta**	**O1 Theta**
**Genotype**	**Control**	21.87±4.26	49.58±6.92	104.52±9.90
	**RTT**	77.44±35.75	124.14±61.28	192.08±79.72

AUCs reported as Mean±SEM

* p<0.05 for differences between RTT and control groups and # p<0.05 for differences within group by age.

### Delta power regression over consecutive SWS cycles overnight was lost in RTT

In addition to the overall heightened delta power, another noticeable difference for the SWS sleep efficiency for RTT was the lack of delta power regression in consecutive SWS cycles noted in all control EEGs. In the control group, the first SWS cycle had the highest delta power of the night. The SWS cycles that followed the first cycle showed a decrement in delta power in consecutive cycles (Fig 2A, compare control to RTT traces). This phenomenon was lost in RTT EEGs. The heightened delta power detected in the first RTT SWS cycle remained heightened over all the consecutive cycles. Repeated measures AVOVA for the first 3 SWS cycles in the control group showed significant within subjects effect for delta power in O1 (p<0.001; F = 13.95). The within subjects effect for delta power in O1for RTT however was not significant for O1 (p = 0.45; F = 0.84). Therefore the physiological reduction of delta power over consecutive SWS cycles in overnight EEGs was lost in RTT. The heightened delta remained heightened all through the night (Fig 2A).

### Rate of rise in delta power within each SWS cycle

To evaluate the dynamics of delta power after the initiation of a SWS cycle using non-traditional codes meant to reflect traditional N1 to N3 NonREM stages with increasing delta power, the rate of rise in delta power was quantitated for each cycle. The rise in delta power over the first 10 minutes in RTT EEG channels was F3 (198.39±57.08%), C3 (220.40±66.87%), and O1 (187.97±63.55%) and control EEG channels was F3 (197.03±21.61%), C3 (196.68±16.60%), and O1 (180.33±14.81%) and were not significantly different between the two groups or along AP axis within each group (repeated measures ANOVAs, within-subjects effect, F = 0.745, p = 0.45 for controls and F = 1.6, p = 0.23 for RTT). The overall rise in delta power reflecting overall change from the traditional N1 to N3 stages during SWS in RTT EEG channels were F3 (569.31±223.54%), C3 (613.49±155.47%), and O1 (449.80±166.53%) and in control EEG channels was F3 (295.15±48.03%), C3 (299.77±34.34%), and O1 (295.02±29.28%) and were not significantly different along the AP axis (repeated measures ANOVAs, within-subjects effect, F = 0.11, p = 0.95 for controls and F = 0.43, p = 0.63 for RTT). Although the RTT groups showed a higher overall rate of rise in delta, the differences were not significantly different by group.

### Temporal evolution of SWS cycles: characteristics by age

**Fig 3:**
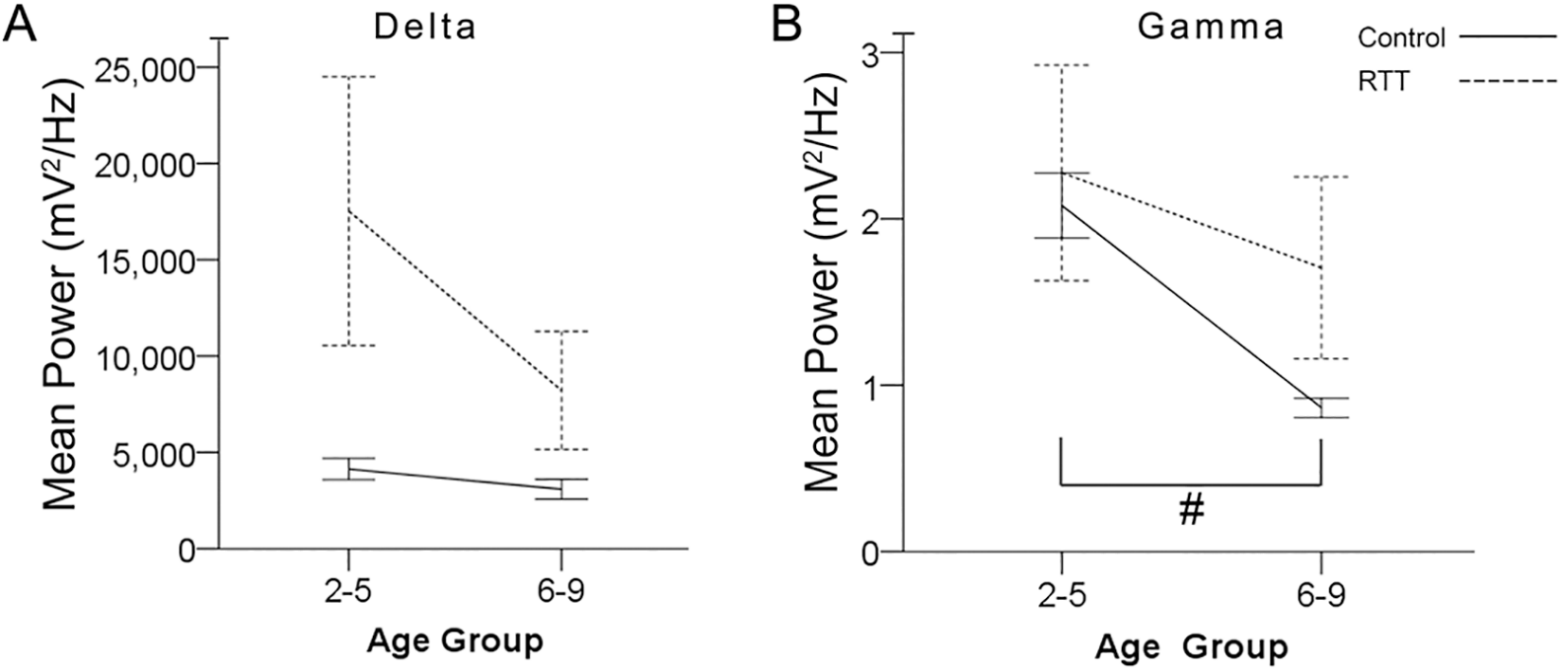
Age-dependent evolution. Because occipital lead displayed the greater difference between genotypes, occipital line graphs were used to display age related comparison. (A) Comparison of delta power revealed no significant difference between ages. Difference in delta power between Control EEGs and RTT EEGs seems to be driven by 2–5 year age group. (B) Gamma power in control group had a significant decrease from age group 2–5 year to 6–9 year group. The sharp decrease in gamma power is lost in patients with RTT as age increases.

Age related temporal evolution of SWS cycles was analyzed by grouping and comparing qEEG data between 2–5 and 6–9 year olds. No significant differences between the numbers of SWS cycles were detected within each group (i.e.; control and RTT) by age. Delta and gamma power AUC showed an age-dependent (Fig 3, Table 4) decrease. This age-dependent decrease was significant for gamma power in the control group [p = 0.001; 2–5 years old (2.45±0.28 mV2/Hz) and 6–9 years old (0.86±0.06 mV2/Hz); Fig 3B, Table 4 #] but was absent in RTT (p = 0.52). Alpha power did not show any temporal changes during SWS nor did the theta/beta ratios. Delta AUCs although significantly higher in RTT for the group as a whole (Fig 2B), were higher but not significantly different from controls at 2–5 years old (p = 0.15) nor at 6–9 years old (p = 0.16) due to large variability within the RTT group (Fig 3A, Table 4). Similarly, gamma power AUC was not significantly different between RTT and controls for each age group. In summary, the heightened delta power detected in RTT was higher in the younger age-group compared to the older age-group (Table 4), however not significantly. The age-dependent decline in gamma power during SWS, that was significant in controls, was lost in RTT.

### SWS percent by age

**Fig 4:**
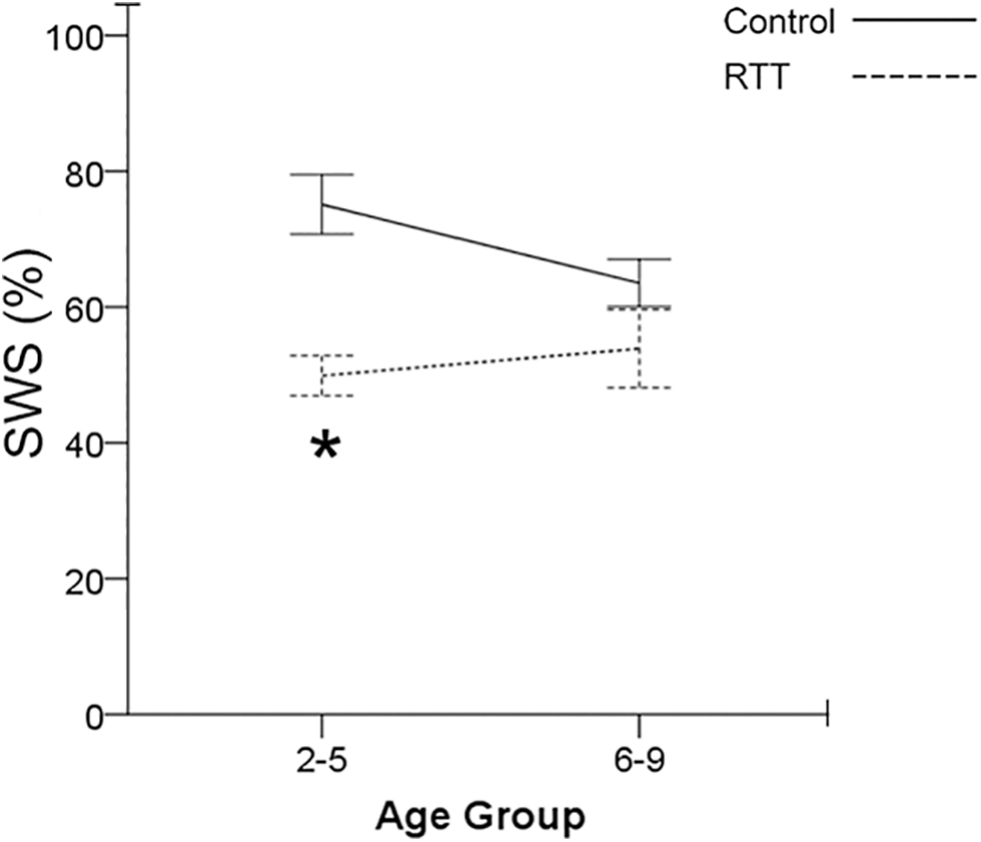
SWS percent in Age Group. Patients with RTT have significantly lower SWS percent compared to control group. Significance is driven in the age group 2–5 year old. The significance in SWS percent is lost in 6–9 years ago. Comparison of SWS percent reveals an increasing tread in SWS percent for patients with RTT instead of the decreasing trend in the control group.

SWS percent, which was defined as the percentage of SWS during each recorded overnight EEG period, was analyzed across age groups and across genotype (Fig 4). Within each genotype group, there were no significant differences for SWS percent between age-group 2–5 yr olds compared to the age group 6–9 yr olds. However, there was a significant difference between genotypes at 2–5 years old. At the younger age the control group’s sleep efficiency (75±4%) was significantly higher (p = 0.001) than the RTT group’s sleep efficiency (50± 3%). This significance across genotype was not detected in 6–9 year olds (p = 0.16; control (64±3%) vs. RTT (54±6%)). Therefore, SWS deficits were driven by the younger age-group in RTT. Additionally, the higher rate of rise in delta power during SWS detected in RTT EEGs that was not significant by genotype-group was significantly higher when analyzed by age-group. The 6–9 yr. old RTT group had an average rate of rise in delta power that was C3 (739.88±118.32%) and significantly higher (p = 0.002) than controls’ C3 (262.88±56.17%).

### Correlations with clinical severity in RTT

**Fig 5:**
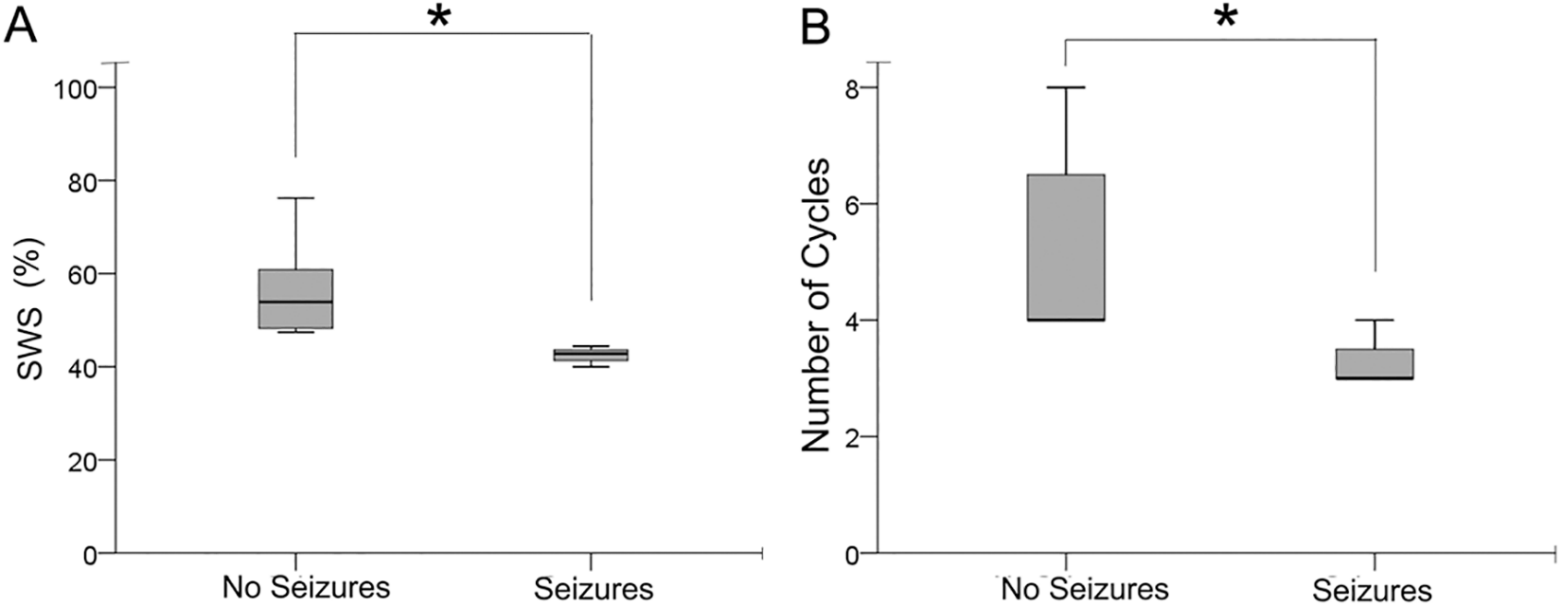
Seizures correlates with high delta power. Clinical severity of patients with RTT were recorded and documented. Seizures are a characteristic of RTT. Patients with RTT were separated into two groups: patients who showed no seizures and patients who experienced seizures (A) Patients with RTT who experienced seizures correlated negatively with lower SWS percent. (B) In addition patients with RTT who experienced seizures correlated negatively with lower cycles during sleep.

As part of the evaluation of every patient before acquisition of overnight EEGs, clinical severities were scored according to a pre-defined scale (Tables 1 and 2; see methods). Scores for individual parameters were added to create a compound severity score (Total SS) score for each patient (Table 2). The EEG reports for the overnight EEGs recorded are listed in Table 3. The epileptiform discharges reported were sharp waves and spike waves which are not known to significantly contaminate EEG spectral analyses in general (Nair et al., 2014). No seizures were recorded in during the overnight EEGs in the RTT group. SWS sleep impairments detected with qEEG showed correlations with some of these clinical scores. Correlation with seizure scores (SZ) calculated over a 1 month period of self-reported data (parents) showed a strong and significant correlation with lower SWS percent (r = -.809, p = 0.005) and fewer SWS (r = -.656, p = 0.039) cycles (Fig 5).

## Discussion

In this study, the most significant qEEG biomarkers during SWS were the heightened delta power in RTT that did not undergo overnight regression like the age-matched controls. Delta and gamma power AUC showed an age-dependent decrease (Fig 3) similar to previous reports (Clarke et al., 2001; Campbell et al., 2012; Baker et al., 2012b).The age-dependent temporal evolution of SWS from ages 2–9 yrs. old in RTT showed significant impairments. Previous studies have reported RTT associated sleep problems [8,12,28]. However, few studies have used qEEG to identify the biomarkers associated with RTT but not specifically for the sleep dysfunction [39,40]. An important goal of this study was to evaluate the translational value of insights gained from qEEG studies in the RTT animal model to provide a better understanding of the impairments in EEGs from girls with RTT. We applied algorithms developed to quantitate sleep dysfunction in 24h EEGs from a Mecp2 KO mouse model^Mecp2tm1.1Bird^of RTT [26,41] to the present human EEG data sets. As anticipated from previous reports, significantly lower SWS percent was detected in RTT overnight EEGs similar to the animal model studies. However, since the RTT clinical EEGs did not have polysomnography data, the study design did not allow us to determine whether the significantly lower SWS percent detected in RTT was due to increased REM or increased wakefulness or both. It is known that RTT is associated with epilepsy and epilepsy can independently and significantly alter SWS sleep [42,43]. This study found a significant positive correlation between the number of documented seizures in patients with RTT and significantly lower SWS efficiencies within the RTT group.

### Unknown role of MECP2 in sleep architecture

The role of *MECP2* in sleep architecture is currently unknown. Animal model studies in male Mecp2 KO mice^Mecp2tm1.1Bird^ revealed significantly blunted delta power during SWS sleep compared to the age-matched WT mice at 7 weeks of age [26]. The same algorithms applied to overnight EEGs from girls with RTT and age-matched controls allowed for comparisons between the findings from the animal model study and the clinical measures. In contrast to the significantly blunted delta detected during SWS in KO male mice ^Mecp2tm1.1Bird^, the girls with RTT who had a variety of *MECP2* mutations (Table 1) with variable expression in brain; consistently showed significantly heighted delta power during SWS at ages 2–9 yrs. It is of interest that the complete absence of MeCP2 in male mice^Mecp2tm1.1Bird^ and partial absence in girls aged 2–9 yrs. with RTT both resulted in significant alteration of delta power albeit in opposite directions. All other spectral powers examined in the murine model and RTT girls during the same SWS cycles remained similar to controls.

Sleep is thought to facilitate a global synaptic downscaling, renewing brain’s capacity to encode new information. Sleep also supports the formation and consolidation of long-term memories [44]. Both the freeing of encoding capacity and memory consolidation are performed during SWS sleep. EEG power in the 0.5–4 Hz band-width is a reliable measure of the number of SWS sleep cycles during sleep [45]. SWS, has also been linked to synaptic homeostasis in the brain. Evidence shows a widespread increasing synchronization in neuronal activity of networks during SWS [44]. Because increased synaptic strength favors neuronal synchronization, SWS reflects the synaptic efficacy in the network [44]. Therefore the developmental changes in SWS power reported in several studies [15–17] has been proposed to be driven by cortical synaptic pruning associated with maturation in developing brains. The heightened delta power during RTT SWS (300%) that was driven by the younger age group in this study may indicate a lack of synaptic maturation during that period. This finding is of interest as it corresponds with the significantly higher expression of glutamate receptors in younger (i.e.; ≤ 8yr old) RTT brains [46] as well as in 2 week old Mecp2 KO male mice^Mecp2tm1.1Bird^ [47]. We would predict that the significantly reduced expression of glutamate receptors detected in older patients with RTT (i.e.; ≥ 8yr old) would be associated with blunted delta power during SWS similar to that reported for the 7 week old Mecp2 null mice [26]. The 7 week old male Mecp2 null mice have been shown to have significantly lower expression levels of glutamate receptors compared to 2 week old male KO mice^Mecp2tm1.1Bird^ [47] and their age-matched WT litter mates. This prediction could explain the reason behind the opposite effects on delta density detected during SWS in the male KO mice vs. the young patients with RTT. Future studies could test the prediction by evaluating 24h qEEGs in 2 week old male mice and girls with RTT who are ≥ 10 yrs. old.

Studies have shown that from early childhood to late adolescence [healthy human subjects (2.4–19.4 years)], the location of maximal SWS activity during sleep shifts from posterior to anterior regions as the brain matures [17,48]. Our automated algorithm identified occipital predominance of SWS in qEEG of RTT and control females below 10 yrs. of age. The shift along the postero-anterior axis has been reported only for the SWS frequency range, which remained stable throughout night sleep [35,49] is specific to young children and switches to frontal predominance in young adults. Longitudinal MRI studies indicate that cortical regions undergo maturational changes at temporally different speeds and sequences [50,51] such that cortical maturation starts early in posterior areas and spreads rostrally to the frontal cortex. Cognitive and behavioral functions associated with the frontal cortex do not mature until late adolescence [50]. SWS shifts reflect these cortical maturational processes and therefore show a similar postero-anterior spatial evolution with higher SWS power in occipital leads (i.e.; similar to our findings) in younger brains that shifts anteriorly as they mature into adolescence. It has therefore been hailed as a marker for plastic changes during childhood, and as a tool to investigate cortical maturation both in health and disease [35]. The findings of heightened delta determined by the automated algorithm shows that the differences among age groups are driven by the younger subjects in the current study, which supports these previous conclusions.

### Progression of sleep disturbances in girls with RTT

Few studies have examined the evolution of the sleep disturbances in RTT with age. Recent reports from studies that investigated the trajectories and influences of age, mutation and treatments in RTT patients from ages 2–35 yrs. have reported differences driven by age and genotype. However, treatment was not associated with improvement in sleep problems [7]. Similar studies investigating qEEG using FFT and power analysis are lacking. The two AED drugs the girls with RTT were taking during this study were Depakote and Keppra (n/n = 4/10; Table 3). Leviteracetam (Keppra) has been reported to have no effect on EEG spectral power (Veauthier et al., 2009; Mecarelli et al., 2004) unlike CBZ/phenytoin and phenobarbital which are known to increase delta and theta power. Additionally, valproic acid has been shown to decrease EEG synchronization in children in a use dependent manner (Clemens, 2008) and shown to decrease delta and theta power.

In this study, age-dependent evolution of the heightened delta in RTT showed that the younger age groups had higher overall delta power during SWS. The trend however was not statistically significant due to a large variability in the RTT group which may be driven by the type of mutation [for e.g.; R168X (P) EEG showed the largest increase in delta power and the R168X (P) mutations in RTT are also associated with the more severe clinical RTT phenotypes [52]]. Age-dependent evolution in gamma power showed a significant decrease with age in control EEGs which was lost in patients with RTT. In humans, gamma waves develop during childhood and peak around 4–5 years old. Findings suggest that high gamma power indicate central adrenergic activity during sleep. Studies have shown that gamma powers during SWS are very similar to gamma responses during tasks performed while wake, which reflects an increased alertness. It has been proposed that gamma oscillations during SWS may reflect recalled events experienced previously [53]. Gamma waves are also involved in epileptiform activity, progressively increasing in frequency from the pre-ictal to the ictal state [54]. The loss of the age-dependent decrease in gamma power during SWS in RTT highlights another potential biomarker underlying dysfunctional sleep that has not been reported before. Its functional significance requires further study.

Delta waves during SWS are thought to reflect the brain reversing the effects of waking. However, sleep deprivation also increases SWS delta power [55] indicating that the declining sleep efficiency commonly reported in RTT, would act to increase delta power in RTT SWS. Additionally, day time sleepiness is commonly reported in RTT and its association with and implications for the night time heightened delta reported here need further study. Previous studies in Mecp2 KO mice^Mecp2tm1.1Bird^ showed a severe impairment in activity dependent glutamate homeostasis [26] associated with significantly higher brain levels of glutamate. Similar findings of high glutamate levels have been reported in patient cerebrospinal fluid samples [56] for ages 1 to 17 yrs. old which also seem to be driven by the younger patients with RTT. The long-term failure of extracellular glutamate homeostasis detected in the RTT mice, was associated with a noteworthy attenuation of delta wave power during SWS sleep. The heightening of delta power during SWS in girls with RTT begs the question of what the glutamate homeostasis response during sleep may be and also indicates a crucial role of delta waves in synaptic physiology both in health and disease [57].

Although no diagnostic EEG patterns in RTT have yet been described, most studies have found progressive deterioration of the EEG with worsening functional impairment [3]. Attempts have been made to develop a staging system for EEG patterns in RTT that would correlate with clinical progression. Our study provides evidence of possible EEG biomarkers that correlate with the clinical severity in patients with RTT. The chronic poor SWS percent by itself may also modulate systemic metabolic functions both in humans and rodents as recently shown [58]. Moreover, the history of seizures was found to be associated with fewer numbers of SWS cycles and lower SWS percent contributing to the vicious cycle.

### Conclusion

This qEEG study demonstrates that in addition to SWS deficits such as fewer SWS cycles, heightened delta power is a unique biomarker of SWS dysfunction in RTT. The similarities of SWS sleep dysfunction detected between the RTT mouse model and RTT patients indicates that investigating mechanisms underlying SWS sleep anomalies in pre-clinical models is a worthwhile endeavor. The findings of this study indicate that non-traditional automated spectral analysis and algorithms developed to quantitate those in animal models can be used as a helpful tool in addition to conventional sleep scoring protocols to quantify impairments in RTT sleep. The qEEG biomarkers may also help in evaluating the efficacy of novel treatments on the quality and evolution of SWS in RTT which may have to be tailored based on age.

## Supporting Information

S1 Fig Raw Traces correlating with low and high delta power. 10 second traces were documented for control group and patients with RTT at both low and high delta power periods. (A) (B) Comparison of control EEGs and RTT EEGs revealed similar raw traces at low delta power. (C)(D) However, comparison of control EEGs and RTT EEGs at high delta power revealed an increase in frequency of delta activity rather than increase in power in RTT EEGs compared to Control EEGs.
(TIF)

S1 Table PSG sleep score data for age-matched control girls. (DOCX)

## Abbreviations

SWS: Slow wave sleep
qEEG: quantitative electroencephalogram
TBR: theta beta ratio
AUC: area under curve
RTT: Rett syndrome
MECP2: methyl-CpG-binding protein 2 gene
REM: rapid eye movement
NonREM: non-rapid eye movement
AP axis: Anterior posterior axis
