# Supplementary figures and images for: Heightened Delta Power during Slow-Wave-Sleep in Patients with Rett Syndrome Associated with Poor Sleep Efficiency
Source: PLoS One. 2015 Oct 7;10(10):e0138113. doi: 10.1371/journal.pone.0138113 (PMC4596813; doi:10.1371/journal.pone.0138113)

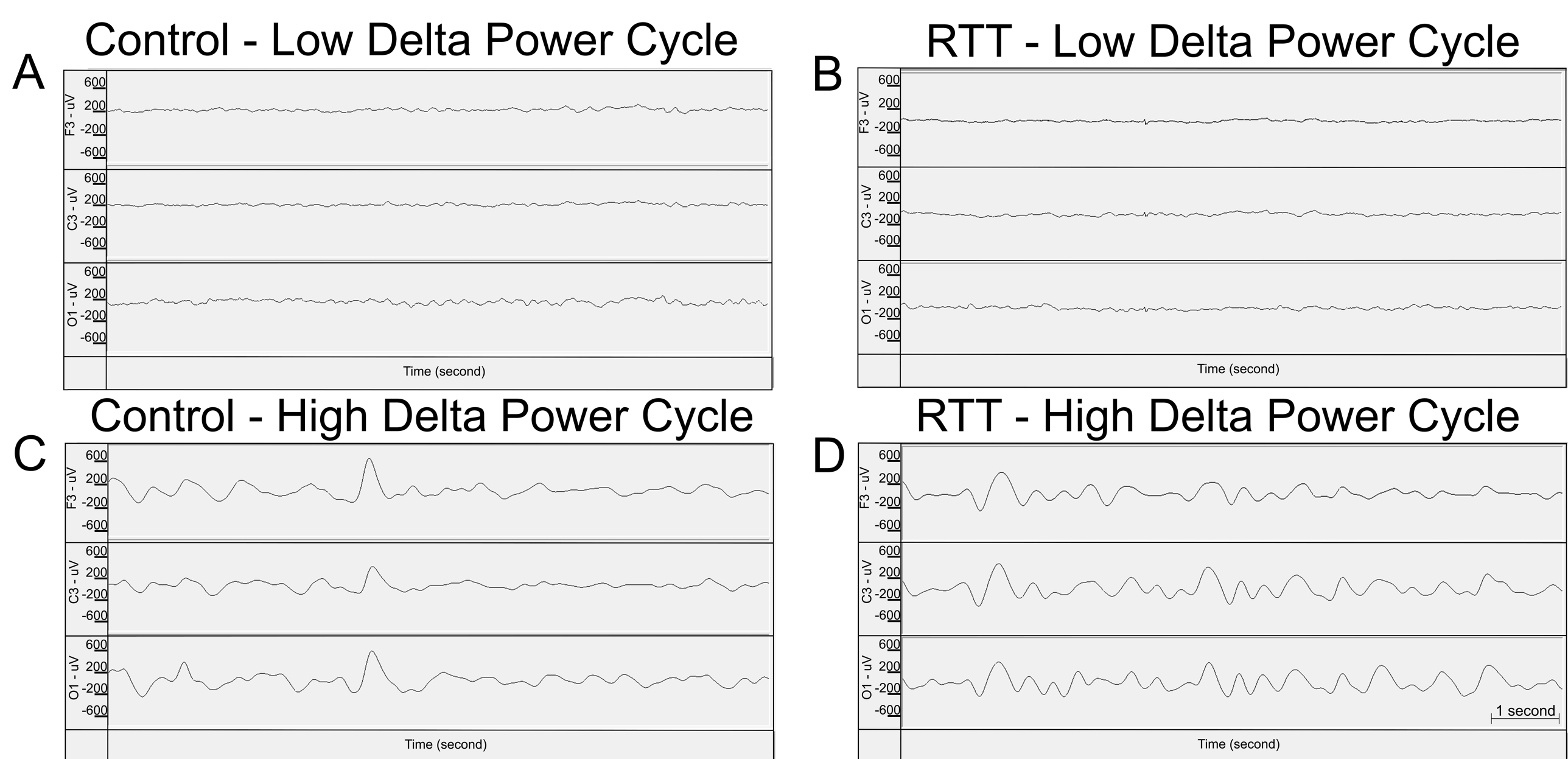

Supplement: S1 Fig — 10 second traces were documented for control group and patients with RTT at both low and high delta power periods. (A) (B) Comparison of control EEGs and RTT EEGs revealed similar raw traces at low delta power. (C)(D) However, comparison of control EEGs and RTT EEGs at high delta power revealed an increase in frequency of delta activity rather than increase in power in RTT EEGs compared to Control EEGs. (TIF) [file pone.0138113.s001.tif]
