# Supplementary material for: Heightened Delta Power during Slow-Wave-Sleep in Patients with Rett Syndrome Associated with Poor Sleep Efficiency
Source: PLoS One. 2015 Oct 7;10(10):e0138113. doi: 10.1371/journal.pone.0138113 (PMC4596813; doi:10.1371/journal.pone.0138113)
Supplement: S1 Table — (DOCX) [file pone.0138113.s002.docx]

| Control ID | Age (yrs.) | Total recording time (min) | Total sleep time (TST)-(min) | Sleep Efficiency (%) | Stage 1 (N1)  (% TST) | Stage 2  (N2) (%TST) | Slow wave sleep (N3) (%TST) | REM (%TST) | Arousal index (N/hr) | Obstructive apnea hypopnea index (N/hr) | SpO2 nadir (%) |
| --- | --- | --- | --- | --- | --- | --- | --- | --- | --- | --- | --- |
| Patient1 | 3 | 505.57 | 434.00 | 86.45 | 4.15 | 32.60 | 34.22 | 29.03 | 16.73 | 0.41 | 90 |
| Patient2 | 3 | 521.43 | 461.00 | 90.57 | 0.76 | 54.12 | 25.81 | 19.31 | 7.55 | 0.13 | 95 |
| Patient3 | 4 | 550.50 | 474.50 | 88.94 | 1.58 | 31.09 | 38.36 | 28.98 | 7.46 | 0.89 | 93 |
| Patient4 | 4 | 570.70 | 464.50 | 83.84 | 2.91 | 35.20 | 33.37 | 28.53 | 13.43 | 1.29 | 92 |
| Patient5 | 4 | 512.78 | 370.50 | 75.61 | 3.64 | 33.20 | 50.61 | 12.55 | 9.07 | 0.16 | 94 |
| Patient6 | 5 | 503.78 | 458.50 | 91.70 | 4.47 | 36.42 | 39.04 | 20.07 | 10.86 | 0.79 | 90 |
| Patient7 | 5 | 521.62 | 454.00 | 91.44 | 1.21 | 39.32 | 32.05 | 27.42 | 8.99 | 0.53 | 94 |
| Patient8 | 6 | 494.82 | 431.50 | 89.80 | 4.52 | 33.95 | 35.92 | 25.61 | 10.15 | 0.28 | 92 |
| Patient9 | 7 | 550.33 | 508.50 | 95.85 | 1.28 | 54.28 | 30.58 | 13.86 | 12.27 | 0.83 | 92 |
| Patient10 | 7 | 457.70 | 404.00 | 89.18 | 7.05 | 34.65 | 35.77 | 22.52 | 12.62 | 0.30 | 92 |
| Patient11 | 7 | 610.32 | 341.00 | 69.66 | 10.56 | 38.71 | 31.82 | 18.91 | 18.83 | 1.06 | 92 |
| Patient12 | 7 | 474.65 | 410.50 | 87.81 | 0.49 | 52.38 | 32.16 | 14.98 | 6.72 | 0.00 | 93 |
| Patient13 | 8 | 559.35 | 531.00 | 95.93 | 1.51 | 42.09 | 31.73 | 24.67 | 7.46 | 0.00 | 93 |
| Patient14 | 8 | 564.57 | 435.00 | 78.59 | 2.76 | 53.22 | 25.17 | 18.85 | 8.00 | 0.28 | 94 |
| Patient15 | 8 | 544.93 | 433.50 | 81.95 | 1.96 | 58.59 | 22.84 | 16.61 | 10.80 | 0.55 | 93 |

Suppl. Table 1. PSG sleep score data for age-matched control girls
